# Ingestion of colostrum from specific cows induces Bovine Neonatal Pancytopenia (BNP) in some calves

**DOI:** 10.1186/1746-6148-7-10

**Published:** 2011-02-18

**Authors:** Annette Friedrich, Mathias Büttner, Günter Rademacher, Wolfgang Klee, Bianca K Weber, Matthias Müller, Annette Carlin, Aryan Assad, Angela Hafner-Marx, Carola M Sauter-Louis

**Affiliations:** 1 Clinic for Ruminants LMU Munich, Sonnenstr. 16, 85764 Oberschleissheim, Germany; 2 Bavarian Authority for Health and Food Safety, Veterinärstr. 2, 85764 Oberschleissheim, Germany; 3 Bavarian Authority for Health and Food Safety, Eggenreuther Weg 43, 91058 Erlangen, Germany

## Abstract

**Background:**

Since 2006, cases of haemorrhagic diathesis in young calves have been observed with a much higher incidence than previously known. The syndrome, now uniformly called Bovine Neonatal Pancytopenia (BNP), is characterized by multiple (external and internal) haemorrhages, thrombocytopenia, leukocytopenia, and bone marrow depletion. Although various infectious and toxicological causes of bleeding disorders in calves have been ruled out, the aetiology of BNP remains unknown. However, field observations have led to the hypothesis that the aetiological principle may be transmitted to calves via colostrum.

The objective of the present study was to verify whether ingestion of colostrum from dams of known BNP calves can elicit signs of BNP and typical haematological findings in conveniently selected neonatal calves. Six such calves received one feeding of colostrum (or a mixture of colostrum batches) from dams of known BNP calves. As controls, another six conveniently selected calves from herds which had never had a BNP case received one feeding of colostrum from their own dams. Haematological and clinical parameters were monitored.

**Results:**

One of the six experimental calves never showed any haematological, clinical or pathological evidence of BNP. In the other five calves, thrombocyte and leukocyte counts dropped within a few hours following ingestion of colostrum. Of those, three calves developed clinical signs of BNP, their post-mortem examination revealed bone marrow depletion. Of the remaining two calves, a pair of mixed twins, marked thrombocytopenia and recurrent leukocytopenia was evident in one, in which only slight changes in the bone marrow were detected, while in the other thrombocyte counts dropped, but rebounded later, and no bone marrow changes were noted. Thrombocyte counts of the experimental calves were statistically significantly lower than those of the control calves at 2 hours post ingestion of colostrum and at every sampling point between 9 hours and 8 days postcolostral. Leucocyte counts of the experimental calves were statistically significantly lower than those of control calves at 2 hours post ingestion of colostrum and 3-7 days postcolostral.

**Conclusions:**

BNP can be induced in some calves by ingestion of colostrum from cows that have given birth to BNP calves.

## Background

During the 20 years preceding 2006, sporadic cases of unexplained bleeding in cattle of various ages, including, in 1989 and 1991, two calves less than four weeks of age with panmyelophthisis, were admitted to our clinic. Starting in 2006, there has been a remarkable surge in cases of bleeding disorder in young calves. In December, 2009, the scientific name "Bovine Neonatal Pancytopenia" (BNP) was adopted for this syndrome because of the characteristic changes in haematological parameters. Other names are "haemorrhagic diathesis", "bleeding calf syndrome" or "blood sweating". The disease is defined by multiple (external and internal) haemorrhages, thrombocytopenia, leukocytopenia, and bone marrow depletion in calves younger than four weeks [1-9]. No consensus exists with regard to presence of BVDV as an exclusion criterion.

While the increase in incidence was first noted in Bavaria, cases have also been recorded in other federal states of Germany, albeit with striking differences in incidence. Other European countries (e.g. United Kingdom, Ireland, Netherlands, Belgium, Luxembourg, France, Italy, Spain) have also reported cases [10,11]. Various known causes of bleeding disorders (e.g. genetic, such as the hereditary platelet disorder in Simmental cattle, toxic causes such as intoxication by bracken fern, infectious causes due to e.g. parvo-virus) have been ruled out [1-3,12-14], but the cause is still unknown. Kappe et al. [14] reported the detection of PCV2-antigen in five of 25 calves with BNP and in one of eight control calves and discussed an aetiological role of this virus, while in the investigations of Mueller et al. [3] and Willoughby et al. [12] PCR testing for PCV2 gave negative results. No cases were reported from countries free of BVD or without vaccination against BVD, e.g. Denmark, Austria, and Switzerland.

The age at first appearance of clinical signs (mean 14 days) and observations by farmers were compatible with an aetiological role of colostrum. Except for one unconfirmed report from Scotland [15] we are not aware of any evidence of thrombocytopenia occurring before colostrum intake.

The objective of this study, therefore, was to investigate the effect of colostrum of cows that had given birth to at least one calf affected by BNP on neonatal calves from farms where the disease had never occurred. The trial was approved by the ethics committee of the government of Upper Bavaria, and was carried out in cooperation with the Bavarian Authority of Health and Food Safety.

## Methods

### Hypotheses and sample size determination

The sample size determination was based on following assumptions and hypotheses:

• The incidence of BNP among the cattle population (an estimated 50 cases among 1 million calves per year would yield a probability of 0.00005 for the chance occurrence of the disease in a randomly selected calf - these numbers originated from the incidence observed in Bavaria at the time of the sample size determination). Therefore the null hypothesis was that the disease could not be reproduced by the feeding of colostrum, but that the disease occurs with a sporadic incidence of 0.00005.

• At the time of the sample size determination, owners of farms with BNP reported that more than half of the cows that had given birth to a calf later affected by BNP had another affected calf in the subsequent year. Therefore the alternative hypothesis stated that the disease can be provoked in at least 50% of the cases by feeding colostrum of cows that had already had an affected calf to neonatal calves of "unsuspicious" dams.

• For the sample size determination a power of 90% was chosen in a one-sided test for one proportion, and four animals were calculated as minimal sample size. Two additional animals were enrolled as reserves in case of unexpected losses.

• Since no information on thrombocyte counts in calves during their first few hours of life were found in the literature, six healthy calves were sampled as controls.

### Dams

The dams of the experimental calves (three Holstein Friesians and two Brown Swiss) stood in one of two large dairy farms where BNP had never occurred. The dams of the control calves were six healthy cows (five Holstein Friesians and one Simmental) that had been purchased by the clinic for a different study from farms where BNP also had never occurred. Dams of all calves used in the experiment (experimental and control calves) were tested for BVDV-antigen (BVDV-Ag ELISA: Idexx) and BVD antibodies (ELISA: Svanovir BVDV Antibody^®^). Tests were performed according to the manufacturers' recommendations. According to their history, as reported by the herd owners and local veterinarians, none had been vaccinated against BVD but all had been vaccinated against Bluetongue in the course of a mandatory vaccination campaign during 2008 and 2009 (Bluevac-8^®^: Boehringer/CZV Veterinaria). The dams of all experimental calves and all control calves, except for calf 8, had detectable BVDV antibodies. BVDV antigen was not detected in any of the samples.

The four dams whose colostrum was used for the experimental calves originated from two farms with multiple cases of BNP since 2006. All four dams had been vaccinated with a particular inactivated BVD vaccine (PregSure-BVD^®^: Pfizer). Both farms started their BVD-vaccination programme for preventive reasons (farm α started in 2005 with PregSure-BVD^®^: Pfizer, farm β started in 2000 with Rispoval^®^: Pfizer and switched to PregSure-BVD^®^: Pfizer in early 2006). In both herds, no evidence of the presence of PI animals had been found on the basis of young stock serology. Therefore, examination for BVDV antigen and BVDV antibodies was intentionally omitted. During 2008 and 2009, these cows were also vaccinated against Bluetongue (Bluevac-8^®^: Boehringer/CZV Veterinaria). Information on these four dams is given in Table 1. Two of the cows (A and B), originating from one farm (α) had already had two calves with BNP each (in 2008 and 2009). The other two cows (C and D) originated from the second farm (β). One (C) had had a case among her offspring in 2008 and her colostrum from 2009 was not used on the farm. The calf born in 2009 of the other dam (D) was a confirmed case of BNP. All colostrum batches originated from the year 2009.

**Table 1:** Information on experimental and control calves and the batches of colostrum fed

Calf (no.)	Breed	Calf from farm	Colostrum from cow	Colostrum from farm	Litres of colostrum ingested
**Experimental calves**					
1	Holstein Friesian (HF)	I	50% **A **(2^nd ^milking)+ 50% **B **(1^st ^milking)	α	3
2	Crossbreed*	I	50% **A **(2^nd ^milking)+ 50% **B **(1^st ^milking)	α	3
3	HF	II	**A **(1^st ^milking)	α	3
4	Crossbreed*	I	**C **(1^st ^milking)	β	3
5	HF (twin of calf 6)	I	25% **C **(1^st ^milking)+ 75% **D **(1^st ^milking)	β	2
6	HF (twin of calf 5)	I	25% **C **(1^st ^milking)+ 75% **D **(1^st ^milking)	β	2
**Control calves**					
7	Crossbreed**	III	From its own dam		3
8	Simmental	IV	From its own dam		3
9	Crossbreed**	V	From its own dam		3
10	HF	VI	From its own dam		3
11	HF	VII	From its own dam		3
12	HF	VII	From its own dam		3

* Brown Swiss × Belgian Blue; ** HF × Simmental; *** HF × Belgian Blue

All colostrum batches used were from 2009 Cows from which the colostrum was used: **A **and **B **had calves with BNP in 2008 and 2009;

**C **had a calf with BNP in 2008; the colostrum of 2009 was not used on the farm, only in the experiment; **D **had a calf with BNP in 2009

### Experimental calves

Six calves born within a 30 hour interval (July 2009) were purchased from two large dairy farms (I and II) where BNP has never occurred previously (and has not occurred since). The calves (see Table 1) were delivered by three (9, 6, and 4 year-old) Holstein Friesian (HF) and two (10 and 3 year-old) Brown Swiss cows. Calves 5 and 6 were mixed twins born by an HF cow. All calves were transported together to a barn close to the clinic one day after birth (calves 1 and 2) or on the day of birth (calves 3 - 6). There, the calves were housed in individual boxes with slatted wooden floors and straw bedding. The barn had not been used for housing cattle for several years.

### Control calves

Six calves (three HF, one Simmental, one HF × Belgian Blue, and one HF × Simmental cross calves) that were delivered by natural parturition in the clinic, except for calf 8, which was delivered by Caesarean section in the clinic, were enrolled as control calves. The calves were kept in the same way as the experimental calves, but in the clinic.

### Feeding of calves

All calvings occurred under supervision so that the calves could not drink colostrum directly from their dams. Colostrum was provided to all calves in nipple buckets.

The experimental calves were fed one meal of colostrum from cows that had had at least one affected BNP calf in the past. Batches of colostrum had been stored at -20°C and shortly before the colostrum was offered to the calves it was thawed and warmed in a water bath. Since not all colostrum samples exceeded three litres, some calves received a mixture of colostrum batches from two different cows. The matching of experimental calves and colostrum batches is listed in Table 1. The first colostrum intake was between 1.5 and 4.5 hours after birth and all calves, except the twins (no. 5 and 6) received the colostrum on their farm of origin. The twins were fed their first meal at the barn close to the clinic. Calves 1 - 4 received three litres of colostrum, and the twins (calves 5 and 6) received two litres of colostrum.

The control calves received three litres colostrum each from their own dams, which was milked at the clinic and offered to the calves within 1 to 3 hours after birth (Table 1).

Following the first meal of colostrum, all calves were fed whole milk three times a day at a daily volume of approximately 12% of body mass. The milk originated from healthy cows milked at the clinic and at a neighbouring farm and was pasteurised at the clinic before feeding. An exception was the second and third meal for calves 1 and 2 of the experimental group that received whole tank milk from their farm of origin. Fresh water, hay, and calf starter were offered free choice daily.

### Blood sampling (all calves)

An intravenous cannula was placed in a jugular vein of the experimental calves, and precolostral blood samples were taken. The cannula was left in place as long as possible, which varied between 3 and 14 days. If it had to be removed, calves were sampled by venipuncture. While the cannula was left in place, the cannula was flushed prior and post blood sampling with sodium-citrate solution, and the first 10 ml of blood sampled were discarded. If no cannula was left, an 18-gauge (1.2 × 40 mm) needle was used to harvest blood directly into an EDTA-sample tube; no Vacutainer^® ^was used. The intervals between sampling and testing varied between 15 minutes and 24 hours (due to the fact that some samples were taken on the farm of origin).

The control calves were bled by venipuncture at exactly the same time points as the experimental calves. The intervals between sampling and testing also varied between 10 minutes and 20 hours (due to the fact that some control calves were born during the weekend).

The times of blood sampling and the analyses performed for all calves (experimental and controls) are listed in Table 2. In addition to haematological and biochemical examinations these samples were tested for pestiviral genome by duplex real-time RT-qPCR [16] and the presence of BVDV antibodies by ELISA (Svanovir BVDV Antibody^®^) (Table 2).

**Table 2:** Times of blood sampling and analyses performed in the experimental and control calves

	Haematology	Serum biochemistry	Panpestivirus genome	BVDV antibodies
**Precolostral**	X	X	X	X
1,2,3,4,6,9,12 h pc*	X			
**24 h pc**	X	X		
**48 h pc**	X	X		X
Daily until day 14**	X			
**After day 14 every 2**^**nd **^**to 3**^**rd **^**day until euthanasia or day 28**	X			

*pc = postcolostral ** with few exceptions due to laboratory shifts

Determination of cell numbers was performed using the semi-automated haematology analyser Sysmex F-820.

### Clinical examination and termination of trial (all calves)

The study was approved by the ethics committee of the government of Upper Bavaria (ref-nr. 55.2-1-54-2531-56-09).

The calves were planned to be observed for four weeks. Each day the calves were examined clinically with special attention being paid to the skin, oral mucosa, and faeces. Body temperature was measured daily. Calves were treated with antibiotics and/or an NSAID as indicated on the basis of clinical findings. Euthanasia was carried out when obvious BNP or other severe diseases (e.g. septic arthritis or purulent omphalophlebitis) developed which did not respond to conservative treatment within three days or if the calves did not drink their meals three times in a row. It was necessary to allow development of the full range of clinical signs, including haemorrhages, in order to demonstrate the role of colostrum in this disease and allow for refinement in future studies.

All calves were sent for post-mortem examination immediately after euthanasia to the Bavarian Authority of Health and Food Safety (Oberschleissheim). Bone marrow of the femur and sternum was used for histological examination.

The control calves were not euthanized due to ethical and economical considerations, but were sold after being observed for four weeks.

### Statistical analysis

Data on thrombocyte and leukocyte counts were analysed in MS Excel (Version 2007) and SPSS (version 18, http://www.spss.com). Due to the fact that statistical analysis was performed with six animals only per group, non-parametric tests were chosen. For comparison of blood values between the two groups Mann-Whitney-U tests were employed. A significance level of p < 0.05 was used. For graphical display the medians and quartiles were used.

## Results

### Laboratory data

In five of the six experimental calves the thrombocyte and leukocyte values dropped within the first three hours after intake of colostrum. Afterwards the courses varied.

**Figure 1:**
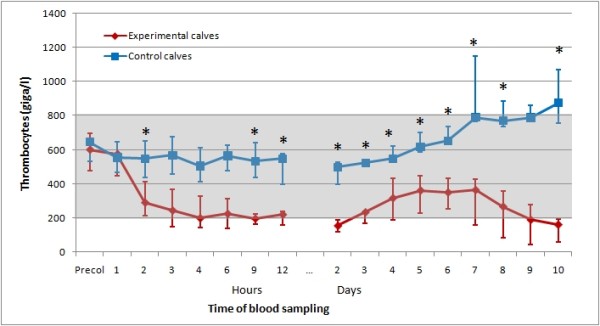
**Medians of thrombocyte counts of experimental and control calves in the study**. Medians of thrombocyte counts of six experimental calves following one feeding of colostrum from specific cows and six control calves fed with colostrum from their own dams. The shaded area represents the reference range; first and third quartile are displayed by error bars, time points with statistically significant differences between the values of experimental and control calves are indicated by *.

**Figure 2:**
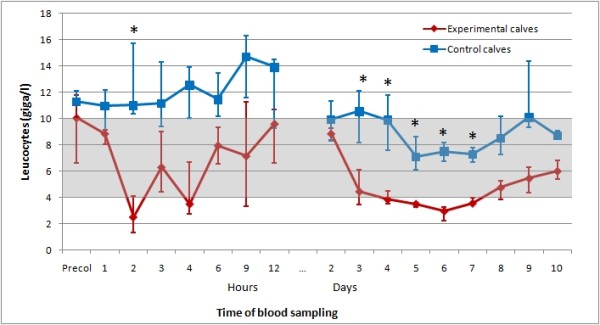
**Medians of leukocyte counts of experimental and control calves in the study**. Medians of leukocyte counts of six experimental calves following one feeding of colostrum from specific cows, and six control calves fed with colostrum from their own dams. The shaded area represents the reference range; first and third quartile are displayed by error bars, time points with statistically significant differences between the values of experimental and control calves are indicated by *.

**Figure 3:**
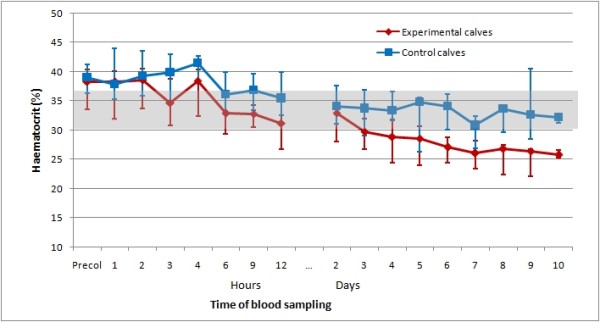
**Medians of haematocrit of experimental and control calves in the study**. Medians of haematocrit of six experimental calves following one feeding of colostrum from specific cows, and six control calves fed with colostrum from their own dams. The shaded area represents the reference range; first and third quartile are displayed by error bars.

For comparison, the courses of thrombocyte and leukocyte counts as well as haematocrit in the experimental and control calves for the first 10 days of the trial are shown in Figures 1, 2, 3.

**Figure 4:**
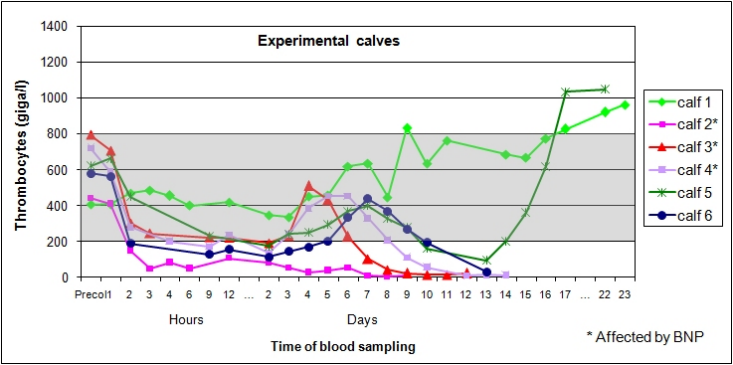
**Individual thrombocyte counts of six experimental calves in the study**. Individual thrombocyte counts of six experimental calves following one feeding of colostrum from specific cows, shown for the whole duration of the study. The shaded area represents the reference range.

Figure 4 shows the courses of thrombocyte counts in all six experimental calves.

The thrombocyte and leukocyte counts of the control calves were never below the respective reference ranges (Figures 1 and 2). The haematocrit in the six experimental calves and six control calves (Figure 3) declined over time, but there was no statistically significant difference at any time between the two groups. The comparison of blood values between experimental and control calves revealed statistically significant (p < 0.05) differences in thrombocyte counts at two hours and at every sampling time from 9 hours up to day 8 and day 10 after colostrum intake, and in leukocyte counts two hours, and every day from day two to day seven after colostrum intake.

Globulin values of the experimental and control calves are presented in Table 3. In all calves a rise in globulin concentration occurred within 24 or 48 h of colostrum ingestion. At no time point statistical significant differences existed between experimental and control calves.

**Table 3:** Globulin concentrations (g/l) of six experimental calves fed with colostrum of specific cows and six control calves fed with colostrum of their own dams.

Calf (no.)	Precolostral	24 h postcolostral	48 h postcolostral
**Experimental calves**			
1	15.0	33.0	29.8
2	15.5	28.4	28.3
3	19.5	n.a.	31.8
4	17.3	20.4	24.1
5	20.3	23.6	19.2
6	17.8	24.0	24.2
**Control calves**			
7	19.9	58.7	n.a.
8	19.6	n.a.	35.6
9	12.8	30.7	30.5
10	15.3	37.9	34.5
11	12.3	17.6	16.9
12	16.9	34.3	31.9

n.a. = not assessed

No pestiviral genome or BVDV antibodies were detected in the precolostral blood samples of the experimental and control calves; but BVDV antibodies were present in postcolostral serum samples from all experimental calves and five of the six control calves. All dams of the experimental calves yielded negative results in the RT-qPCR for the presence of pestiviral genome, but had antibodies against BVDV in the serum. The dams of the control calves also yielded negative results in the examinations for panpestiviral genome and in five of the six cows, BVDV antibodies were detected in the serum.

### Clinical findings

All experimental calves were treated with antibiotics (cefquinome or tulathromycine) and an NSAID (meloxicam) as indicated on the basis of clinical findings.

Only pertinent findings are reported.

**Calf 1 **had small amounts of blood in the faeces intermittently on 9 days (day 4, 6, 8, 12, 13, 15, 16, 19, 22), but did not show any other signs of a bleeding disorder (such as petechiae or external haemorrhages). It had short episodes of diarrhoea on days 2 to 4, and on day 14. On these days the behaviour of the calf was dull. The body temperature was never higher than 40°C but between 39.5 and 40.0°C on 8 days (6, 9, 10, 12-14, 16, 19). The intravenous cannula was removed on day 8, but thrombophlebitis developed nevertheless. This calf was euthanized on day 23 because of severe unilateral gonitis with obvious lameness.

**Calf 2 **had diarrhoea on day 3. On this day and the day before, the behaviour of the calf was dull. On day 4 the faeces contained a small amount of blood as judged by visual inspection and the amount increased daily. Starting on day 6, petechiae could be seen. The body temperature was higher than 39.5°C only on day 8 (39.6°C). The intravenous cannula was fully functional until euthanasia without development of thrombophlebitis. The calf was euthanized on day 9 because of clinical signs consistent with the development of BNP.

The behaviour of **Calf 3 **was dull on days 2 and 9. A moderate navel infection began on day 4. On days 3, 5, and 6 the faeces had small amounts of blood as judged by visual inspection; the amounts increased from day 8 onward. Starting on day 9, petechiae could be seen and they increased in intensity each following day, and starting on day 10, the calf showed prolonged bleeding. The body temperature was higher than 39.5°C only on day 7 (39.7°C). The intravenous cannula was removed on day 4; the calf developed a moderate thrombophlebitis. It had to be euthanized on day 12 because of clinical signs consistent with the development of BNP.

**Calf 4 **had diarrhoea on day 3. During the first 3 days, its behaviour was dull. A moderate navel infection began on day 4. On days 5 and 9, the faeces had a small amount of blood as judged by visual inspection and the amount increased daily beginning on day 11. Starting on day 10, petechiae could be seen. Prolonged bleeding and haematomas were seen on days 13 and 14. The body temperature was never higher than 40°C but between 39.5 and 40.0°C on 4 days (9, 11-13). The intravenous cannula was removed on day 7 because of minimal alteration of the vein, no thrombophlebitis developed. The calf was euthanized on day 14 because of clinical signs consistent with the development of BNP.

**Calf 5 **had very small amounts of blood in the faeces as judged by visual inspection on days 4 to 14, 16 and 18, but did not show any signs of a bleeding disorder. It had short episodes of diarrhoea on days 3 and 18. Its behaviour was dull on days 1, 9, 10 and 19. The calf developed omphalitis on day 3, which resulted in obvious omphalophlebitis on day 20 in spite of antibiotic and antiphlogistic treatment. The body temperature was higher than 40°C on day 16 (40.2°C) and between 39.5 and 40.0°C on 7 days (8-10, 13-15, 18). The intravenous cannula was removed on day 5, but a thrombophlebitis developed nevertheless. This calf was euthanized on day 22 because of severe unilateral carpitis with obvious lameness.

The behaviour of **Calf 6 **was dull on the first two days. An omphalophlebitis began on day 5 and grew worse from day to day in spite of intensive antibiotic and antiphlogistic treatment. On day 11 the faeces contained a small amount of blood as judged by visual inspection. On this day, the amount of blood in the faeces was increased. On days 6 to 8 and 13, petechiae could be seen. The body temperature was higher than 40°C on two days (10, 11) and between 39.5 and 40.0°C on 2 days (12, 13). The intravenous cannula was removed on day 2. The vein remained intact. On day 13 the calf was euthanized because of omphalophlebitis.

The control calves never showed any clinical signs (except for brief episodes of diarrhoea).

### Gross pathology and histopathology

**Figure 5:**
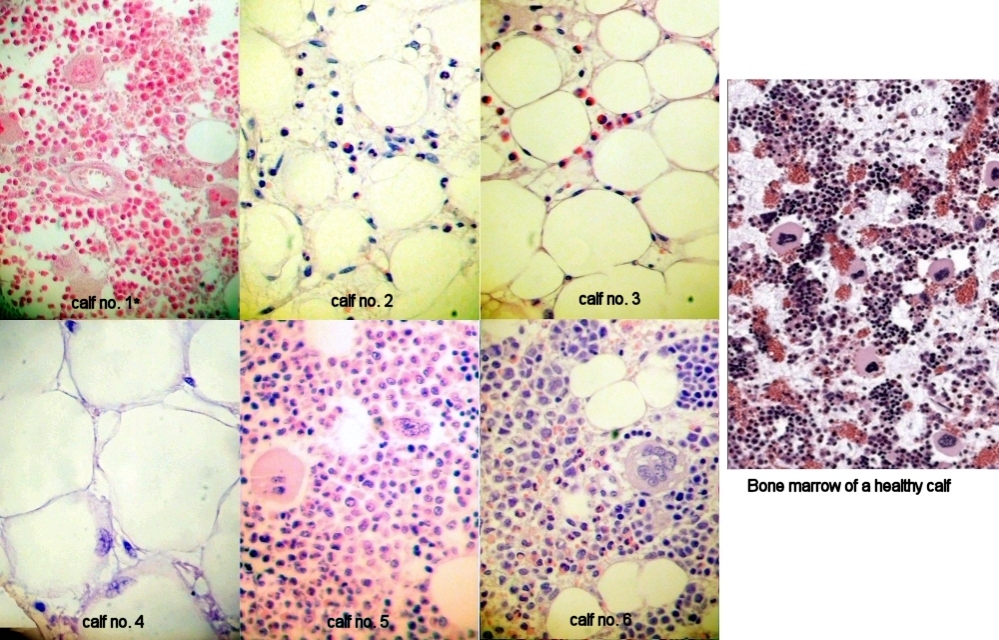
Bone marrow sections of the femur* of experimental calves and one healthy calf. Bone marrow sections of the femur of six experimental calves following one feeding of colostrum from specific cows and one healthy calf. * The picture of calf 1 is taken from the sternum

**Calf 1 **had few petechiae on the ventral aspect of the tongue, pulmonary pleura, and on the thymus, but no overt haemorrhages. Septic gonitis was confirmed. Bone marrow histology was normal (Figure 5).

**Calf 2 **had multiple and extensive internal and gastrointestinal haemorrhages and haematomas, only few megakaryocytes were found in the bone marrow of both femur and sternum, whereas the numbers of other precursor cells were only moderately reduced (Figure 5).

The gross pathology of **calves 3 and 4 **was similar to that of calf 2. In the bone marrow of both femur and sternum no megakaryocytes and only few precursor cells were found (Figure 5).

The clinical findings of omphalophlebitis, septic carpitis and thrombophlebitis in **calf 5 **were confirmed. Few petechiae were detected on the ventral aspect of the tongue and in the pharynx. Bone marrow histology was normal (Figure 5)

The clinical finding of severe omphalophlebitis in **calf 6 **was confirmed. Small haemorrhages on the thymus, abomasal serosa, and in the mesenterium were present. In the bone marrow of both femur and sternum, precursor cells and megakaryocytes were present, albeit in reduced numbers (Figure 5).

Except for calf 3, various bacteria species were found in several organs of all calves.

## Discussion

### Choice of colostrum

As not all batches of colostrum supplied by various farmers had a volume exceeding three litres, a choice had to be made between feeding a homogenous mixture of different batches and feeding different colostrum batches to different calves. The obvious advantage of the first option would have been standardisation of the colostrum offered to the calves. This approach would have required thawing and thorough mixture of a number of batches of colostrum, followed by re-freezing and re-thawing for use. This might have damaged the (unknown) aetiological principle. Another possible disadvantage would have been the dilution of the aetiological agent below a threshold of effect. Because of this, it was decided to feed different batches of colostrum to the individual calves, where possible. In the case of the twins (calves 5 and 6), there were two batches of colostrum of three and one litre left. It was decided to feed them equal volumes; hence these two batches were mixed. The fact that calves 1 and 2 received a mixture of two colostrum batches was due to a misunderstanding but in retrospect proved to be very valuable (see the section on aetiology and pathogenesis).

### Laboratory data

In the reviewed literature only very few papers deal with normal haematology of very young calves, and either report no precolostral samples nor the exact times post colostrum [17] or only indicate an interval during which samples were taken [18-20]. However, none of the articles report values below the reference ranges used here. As demonstrated by the values obtained from the control calves, it is unlikely that the observations reported in the experimental calves are normal occurrences in neonatal calves.

Except in calf 1, the courses of thrombocyte counts show a similar pattern in all experimental calves: there was an initial drop within a few hours after colostrum intake, followed by a temporary rise for a few days, and a final drop to extremely low levels in calves 2, 3, 4, and 6. Calf 5 made a remarkable recovery. On the basis of an average lifespan of thrombocytes of 5 to 9 days [21], about 10 to 20% of thrombocytes can be expected to be removed and replaced per day, or roughly 0.5 to 1% per hour. Thus, even sudden and complete cessation of thrombocyte release from bone marrow could not explain the drop in peripheral thrombocyte counts recorded in the experimental calves in this trial. Hence peripheral destruction must be the main cause.

The drop in thrombocytes cannot be explained by differences in handling following blood sampling. Furthermore, the time between sampling and testing did not affect the number of thrombocytes notably. During a pre-trial blood was sampled and examined at different points up to 72 hours post sampling. There was no notable change in the values obtained. Also the repeatability was evaluated by testing the same blood samples 10 times in a row. There again, no notable differences were found. Therefore it is concluded that the values obtained for the thrombocytes and leukocytes in this study were not influenced by the methods applied. It is also highly unlikely that this drop was caused by transportation of the calves, as the drop was already seen in the first hours after ingestion of colostrum, which was before transportation in calves 1 - 4.

The undulating course in leukocyte counts (Figure 2) may reflect initial peripheral destruction and transendothelial migration, subsequent recruitment, and final depletion due to bone marrow damage. The extremely low levels in affected calves clearly show that both mononuclear cells and granulocytes are affected. Again, no such pattern was seen in the leukocyte counts of the control calves (Figure 2).

### Clinical findings

Most of the experimental calves were somewhat subdued during the first few days. It is assumed that this was due to the transport to the barn on a hot summer day, and the change in environment.

A remarkable finding was that despite the evidence of generalised bacterial infections in five of the six calves, there were only three days on which one of two calves (calves 5 and 6) had a body temperature above 40°C. Therefore, fever does not seem to be a consistent characteristic of BNP. This is in contrast to findings described earlier [1,22].

The first clinical signs in calf 2 were blood in the faeces (day 4) and petechiae on day 6. Calves 3 and 4 showed similar clinical courses as calf 2. They had distinct amounts of blood in the faeces on days 8 and 11, and petechiae on days 9 and 10, respectively. Compared with data given in the literature (average age of 16 days [13] or 17 days [14]) the clinical signs of the bleeding disorder in the experimental calves of this study occurred much earlier. This may be explained by the fact that the calves in this study were examined daily, with special attention being paid to any signs of bleeding disorder, whereas the cases described in the literature may have been detected at later stages of the disease.

Calves 1 and 5 did not develop unequivocal clinical signs of BNP, but had small amounts of blood in the faeces on several days. The postcolostral rise in globulin levels indicates satisfactory resorption of colostrum in all calves. Therefore, failure of resorption cannot be the reason for the differences in the clinical and haematological outcomes.

The high incidence of processes associated with local or systemic bacterial infections in the experimental calves, requiring intensive treatment, despite isolated housing needs an explanation. Impairment of both local and systemic resistance due to the leukopenia observed seems to be the most plausible reason. Whether calves exposed to the aetiological agent develop either BNP or systemic infections cannot be decided on the basis of available data.

### Gross pathology and histopathology

The histological findings in the bone marrow of the six experimental calves indicate that the disease is not an all-or-nothing phenomenon; instead, there can be gradual differences. As Figure 5 shows, there are distinct differences in the bone marrow histology of the six experimental calves. Bone marrow histology of calves 1 and 5 was judged normal, while the bone marrow of calves 3 and 4 showed distinct features of BNP, with no megakaryocytes and only few precursor cells being left. Findings in calves 2 and 6 were not as clear-cut. Although the bone marrow of calf 2 was also judged as being indicative of BNP, the bone marrow of both femur and sternum was not as severely affected as in calves 3 and 4; there were few megakaryocytes left, and other precursor cells were only moderately reduced. Calf 6 showed the least changes, with both megakaryocytes and precursor cells present, but in reduced numbers. Therefore, it cannot be excluded that this calf was affected by BNP, but either at an early stage or in a less severe form. It is not known if such a less severe form of the disease exists, but there is anecdotal evidence that some affected calves do survive the disease.

The control calves were not euthanized due to ethical and economic considerations, therefore no post-mortem examinations or bone marrow examinations are available for these calves. However, their blood values never showed any indication of disturbed production of blood cells. Therefore, it is unlikely that these calves would have shown any bone marrow changes.

In summary, the clinical, haematological and post mortem findings in three of the six experimental calves clearly met the defining characteristics of BNP [1-3,5-9,13]. Whether the thrombocytopenia and blood admixture in the faeces on calf 6 on the day of euthanasia heralded BNP or were consequences of septicaemia remains open to speculation. The findings of bone marrow histology in this calf can be explained by both processes.

### Speculations on aetiology

The fact that calves of various breeds have been simultaneously affected in Europe [13-15,23], as well as among the experimental calves of this study all but rules out an exclusively genetic cause of the syndrome.

Most other possible causes of bone marrow depletion in young calves have been excluded [1]. In view of the very short interval between colostrum intake and changes in blood values, any infectious agent transmitted via colostrum would have to have very unusual properties. Also, a cell-mediated mechanism seems unlikely, as the colostrum batches had been stored at -20°C for at least two months. This mainly leaves toxins and antibodies as candidates for aetiologic principles. (Low molecular) toxins would be expected to cross the placental barrier during pregnancy. Therefore, haematological changes would be expected in precolostral calves. All references in the literature, except for one [15] describe no changes in precolostral calves. One possible hypothesis currently being discussed is an immunopathological reaction due to antibodies - which could be transmitted via colostrum. Many research groups are working on identifying these specific antibodies in the colostrum of cows that have had affected calves. To our knowledge, no specific antibodies have been identified that are linked to the disease. However, all cows whose colostrum was used had been vaccinated against BVD with the vaccine PregSure-BVD^®^, while none of the dams of the control calves had been vaccinated against BVD. This vaccine was taken off the market in Germany in April, 2010, and in all of Europe in June, 2010, by a voluntary decision of the company. It might be possible that vaccination with this vaccine induces the production of specific antibodies which are transmitted via colostrum to the calves and get absorbed and than bind to both peripheral blood cells and stem cells in the bone marrow of the calves. However, these are speculations, and no evidence has yet been published in the literature.

As no precipitous drops in the erythrocyte counts nor signs of haemolysis were recorded, peripheral destruction of erythrocytes does not seem to be a component of the syndrome. The long lifespan of erythrocytes (100 days) [24] explains the relatively slow decline in haematocrit despite bone marrow depletion of all precursor cell lines.

According to the history given by the owners of the dams of the experimental calves and the owners of the dams of the control calves, none of the cows had been vaccinated against BVD; yet all the former and five out of six of the latter group had antibodies against BVD, indicating previous natural infection. In view of the high prevalence of BVD-seropositive animals among the cattle population, this is not surprising, and offers no explanation for the development of BNP in experimental calves.

The aetiological role of colostrum seems to be confirmed by the results of this study. The data presented in Table 1 indicate that all batches of colostrum used had the potential to cause BNP. The colostrum donor cows were selected specifically because they had already had at least one calf affected by BNP. This may explain the high proportion of calves that reacted to the ingestion of colostrum seen in this study. Even with under-detection and under-reporting of cases, the incidence of BNP in the field is relatively low. Therefore, it must be assumed that only few cows have the aetiological principle in their colostrum. Some individual predisposition can also be speculated to be present in the calves. This is evidenced by calves 1 and 2: each received three litres of the same mixture of colostrum batches from two cows. Since the calves were born within about half an hour of each other, the intervals between birth and ingestion of colostrum were comparable. Also, postcolostral globulin concentrations in serum were similar (Table 3). Thrombocyte counts in calf 2 dropped below the reference range within two hours after colostrum intake and subsequently never increased. This calf was the first one to develop signs of BNP and had to be euthanized on day 9. By contrast, calf 1 showed neither a drop in thrombocyte counts nor unequivocal signs suggestive of BNP. Therefore, it seems that the specific colostrum is not pathogenic for all calves, but possibly only to calves with an appropriate individual predisposition.

Some results of calves 5 and 6 also suggest that BNP may not be an all-or-nothing phenomenon. Furthermore, personal observation of patients in our clinic shows that some calves recover from overt disease, and some calves are affected subclinically, showing reduced blood values of thrombocytes and leucocytes, but no clinical signs of BNP (like calf 5). Whether the severe infectious processes that are a plausible explanation for the episodes of moderate fever, and necessitated euthanasia in calves 1, 5 and 6 were due to the lack of immune cells remains unclear. An alternative explanation for the fever would be massive cell destruction. The fact that the calves did not receive colostrum from their own dams may also have contributed to this course of events.

Since the haematological changes occurred well before any treatments were administered, any influence of treatments on the course of events seems very unlikely.

## Conclusions

The results of this study demonstrate that BNP can be reproduced in some neonatal calves by ingestion of colostrum from individual cows that are somehow predisposed to the disease. The available evidence is best explained by the presence in colostrum of a non-cellular, non-infectious agent that leads to a dramatic decline in peripheral blood cell counts (thrombocytes and leukocytes) and all precursor cell lines in bone marrow until complete cell depletion.

## Authors' contributions

Conception and design of the study by AF, MB, GR, and WK; clinical examinations by AF, GR, AC, and AA; post-mortem examinations and bone marrow histology by BKW, MM, and AHM; statistical analyses by CSL and AF; draft by AF, GR, WK, and CSL. All authors read and approved the final manuscript.
